# The management of multi-morbidity in elderly patients: Ready yet for precision medicine in intensive care?

**DOI:** 10.1186/s13054-021-03750-y

**Published:** 2021-09-10

**Authors:** Michael Beil, Hans Flaatten, Bertrand Guidet, Sigal Sviri, Christian Jung, Dylan de Lange, Susannah Leaver, Jesper Fjølner, Wojciech Szczeklik, Peter Vernon van Heerden

**Affiliations:** 1 Department of Medical Intensive Care, Hadassah Medical Center and Faculty of Medicine, Hebrew University of Jerusalem, Jerusalem, Israel; 2 Department of Anaesthesia and Intensive Care Medicine, Haukeland University Hospital, Bergen, Norway; 3 Service de Reanimation, Hopital Saint-Antoine, Paris, France; 4 Department of Cardiology, Pulmonology and Vascular Medicine, Faculty of Medicine, Heinrich-Heine-University Duesseldorf, Duesseldorf, Germany; 5 Department of Intensive Care Medicine, University Medical Center, University of Utrecht, Utrecht, The Netherlands; 6 Department of Adult Critical Care, St George’s University Hospitals NHS Foundation Trust, London, UK; 7 Department of Intensive Care, Aarhus University Hospital, Aarhus, Denmark; 8 Center for Intensive Care and Perioperative Medicine, Jagiellonian University Medical College, Kraków, Poland; 9 General Intensive Care Unit, Department of Anesthesiology, Critical Care and Pain Medicine, Hadassah Medical Center and Faculty of Medicine, Hadassah University Hospital, Hebrew University of Jerusalem, Jerusalem, Israel

**Keywords:** Multi-morbidity, Very old intensive care patients, Time-limited trial

## Abstract

There is ongoing demographic ageing and increasing longevity of the population, with previously devastating and often-fatal diseases now transformed into chronic conditions. This is turning multi-morbidity into a major challenge in the world of critical care. After many years of research and innovation, mainly in geriatric care, the concept of multi-morbidity now requires fine-tuning to support decision-making for patients along their whole trajectory in healthcare, including in the intensive care unit (ICU). This article will discuss current challenges and present approaches to adapt critical care services to the needs of these patients.

## Introduction

Multi-morbidity is defined as the co-occurrence of multiple, usually two or more, chronic conditions in an individual [1]. There have been several attempts to establish criteria for the conditions which qualify for this count. These initiatives range from refining the set of eligible conditions and including the number of affected body systems to considering patterns of recurrences and deterioration [2, 5]. Pearson-Stuttard et al*.* [6] have suggested multi-morbidity metrics based on the onset and sequence of diseases and the clustering of conditions. However, none of these new approaches have been universally accepted so far.

The combination of certain diseases can trigger super-additive interactions [7] resulting in an enhanced effect on functional abilities, quality of life as well as life expectancy and, eventually, may create complex health needs [8, 9]. This especially affects old individuals with an age-related decline in organ function and increase of vulnerability to stress even in the absence of multi-morbidity [10]. Since advanced age is the most important risk factor for multi-morbidity, the prevalence of multi-morbidity is close to 90% in patients aged 85 years or older [11]. Previously devastating and often-fatal diseases have been transformed by modern medicine into chronic conditions. Since the longevity of the population is also increasing, these developments are turning multi-morbidity into a major challenge in the world of critical care [12]. Even in intensive care units (ICUs) designed to manage single-organ conditions, such as cardiac/coronary ICU's, multi-morbidity has become highly prevalent and an important contributor to outcome prediction [13].

After many years of research and innovation, mainly in primary and geriatric care, the concept of multi-morbidity now requires fine-tuning to support decision-making for patients along their whole trajectory in healthcare. Critical care medicine has a particular need for rapid improvement and development since it is mostly organ/system-centred, with survival being the main outcome measure. The holistic view needed for multi-morbid elderly patients, and their individual requirements, still remains a work in progress [14]. During the COVID-19 pandemic, the National Institute for Health and Care Excellence in the UK had to recommend a more holistic approach beyond single scores for organ failure or frailty for deciding about admission to critical care. In particular, it advised that comorbidities and underlying health conditions should be considered when assessing the potential benefit of critical care for the individual patient [15]. This article will discuss challenges and present approaches to integrate multi-morbidity into the decision-making processes in critical care.

### What does multi-morbidity mean in the critically ill patient?

**Fig. 1:**
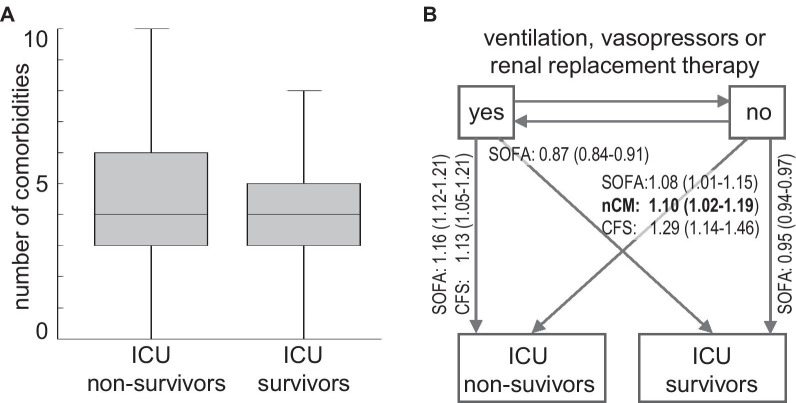
Multimorbidity and ICU outcome in 2103 patients aged 80 years or older from the VIP2 study cohort [16] who were admitted to ICU for more than 24 h and did not have limitations of life-sustaining treatment. Among those, 1455 patients received invasive ventilation, vasopressors or renal replacement therapy. (**a**) Box plot of the number of chronic comorbidities for ICU survivors (n= 1805) and nonsurvivors (n = 298). Logistic regression did not show a significant association with ICU outcome. (**b**) Multi-state modeling with multimorbidity, frailty and baseline SOFA score as covariates [17]. The panel depicts transitions and significant hazard ratios (95% confidence intervals) determined for each point of increase of the number of comorbidities (nCM), clinical frailty scale (CFS) and SOFA score. The number of comorbidities is associated with transition from low-intensity care to death in ICU indicating a role of chronic organ impairments for outcome at that stage.

Multi-morbidity is heterogeneous in phenotype and outcome. There still is no universal concept that describes the burden and impact on individual patients and which would thereby provide useful and actionable information for critical care patients [1]. Merely counting the number of chronic conditions appears too simplistic in this regard. For example, an individual with well-controlled hypertension and osteoporosis is currently considered to be multi-morbid (i.e. by having two chronic conditions), as is someone with end stage chronic kidney disease and chronic obstructive lung disease requiring home oxygen. Clearly, these two situations are not equivalent in terms of prognosis or level of care required. Moreover, some conditions prevalent in older patients, such as sarcopenia or chronic pain, do not influence the early treatment of critical illnesses, although their consequences, e.g. difficult weaning, may necessitate later consideration in their care pathway. Recent findings from the second Very Old Intensive Care Patients study (VIP 2) [16] illustrated the problem of distinguishing chronic conditions, which affect the outcome of critical diseases, from those that do not (Fig. 1A) [16]. However, a more detailed analysis of patient trajectories in ICU suggests that the number of comorbidities still has a role for predicting the clinical course in subgroups of patients (Fig. 1B) [17].

To reflect the quantitative role of individual conditions on the overall impact of multi-morbidity, Min et al. weighed the contribution of each condition with its specific survival rate [18]. This new index outperformed biomarkers of acute physiology for predicting mortality in 440 000 older ICU patients. Moreover, a registry study of 230 000 ICU patients in Denmark suggested that detailed data from the patient's medical history can improve mortality prediction which, however, performs best when combined with characteristics of the acute physiology of organ failure [19]. In a systematic review, Stirland et al. investigated 35 different multi-morbidity indices [20]. Only a minority of these indices passed the authors' threshold for usefulness with respect to the prediction of hospital admissions or mortality. This leaves the essential topic of conceptualizing multi-morbidity for clinical practice open for further research.

### Phenotyping multi-morbidity by clustering diseases

Although multi-morbidity is heterogeneous, the co-occurrence of diseases is mostly non-random and organised in clusters (Table 1). This is obvious for conditions that share the same pathophysiology, such as cardiovascular disorders. For other clusters, the knowledge about the joint pathogenesis is incomplete, such as in cardio-renal syndromes [21]. Almagro et al. recently described gender-specific clusters with neurological and osteoarticular conditions being more frequent in women, while respiratory disorders dominated in men [22]. New clusters can emerge due to exposure to new treatments, e.g. immune checkpoint inhibitors.

**Table 1:** Multi-morbidity clusters in old patients and associated MODS risk profiles

Known condition	Associated/(hidden) conditions	MODS risk profile
Hypertension	CHF, metabolic syndrome	AKI, arrhythmia, stroke
Coronary disease	CHF, CKD, carotid stenosis, PVD	MI/CHF, arrhythmia, stroke, AKI
COPD	Pulmonary hypertension	RVF, arrhythmia, AKI
Diabetes	Coronary disease, PVD, CKD	MI/CHF, AKI, stroke, infection, gastroparesis
Geriatric conditions (examples)		
Frailty	CHF, CKD, sarcopenia, dementia, sensory impairment	AKI, weaning failure, delirium, pressure sores, malnutrition
Polypharmacy	CKD, arrhythmia, coagulopathy, electrolyte disturbances	AKI, CHF, intracranial bleed, delirium
Chronic pain	Osteoporosis, sarcopenia and immobility masking coronary artery disease / CHF, polypharmacy	AKI, HF, delirium, malnutrition

AKI: acute kidney injury; CHF: congestive heart failure; CKD: chronic kidney disease; COPD: chronic obstructive pulmonary disease; HF: heart failure; MI: myocardial infarction; MODS: multiple organ dysfunction syndrome; PVD: peripheral vascular disease; RVF: right ventricular failure

Data-driven research demonstrated that different multi-morbidity clusters are associated with different outcomes in critical care [23]. In addition to predictive modelling, stratifying patients by multi-morbidity patterns would enable targeted interventions in a similar way as single conditions benefit from a more precise understanding of disease phenotypes [24]. In individuals associated with an arteriosclerosis cluster, for example, early adjustment of haemodynamic management might be necessary to protect organs that are at an increased risk for malperfusion, but without detectable dysfunction on presentation. Also important are safety issues which arise when organ-specific therapies can cause collateral damage in other organs with chronic impairments [12]. Eventually, this new approach may give rise to "cluster medicine" which considers multi-morbidity as a set of mostly predictable clusters due to common genetic or environmental pathways [25]. This way of thinking could also change the diagnostic process—a condition that has not yet been diagnosed, but is known to be part of a cluster identified in an individual, is considered present until proven otherwise. For example, critically ill patients with diabetes and long-standing hypertension may need urgent investigation for cardiac conditions to guide fluid resuscitation.

Critical care is embedded in a data-rich environment providing a continuous flow of clinical data. This necessitates the rapid detection of distinct phenotypes of acute diseases, notably in those with substantial heterogeneity. This heterogeneity is partly caused by the presence of co-morbidities [26]. In this context, "cluster medicine", instead of being a nebulous concept, may be seen as an implementation of precision medicine for multi-morbid individuals in ICU. If sufficiently informative data about pre-existing conditions and their association with specific clusters become available, this paradigm could enable prognostication and management of these patients with a high degree of precision.

### Managing multi-morbidity

Management of multi-morbidity is difficult. A recent meta-analysis of various intervention strategies in primary care found only small differences in clinical outcome. Critical illness adds another layer of complexity. Multi-morbidity and the often-associated polypharmacy alter the clinical presentation of many critical conditions. These problems may delay their detection, e.g. sepsis in patients receiving beta-blockers and paracetamol, and interfere with interventions, e.g. fluid resuscitation in congestive heart failure and chronic kidney disease. Of note, the dynamics of some multi-morbidity patterns are contrary to logical expectations, which are based on assuming independence of the underlying conditions. This is shown by the lower mortality in obese individuals with sepsis compared to non-obese patients [27].

Since multi-morbid patients have been frequently excluded from clinical trials, there is a paucity of evidence and guidelines to manage organ dysfunction in these patients [28]. Importantly, applying recommendations devised for the treatment of single conditions could be confusing or even detrimental in this setting [29]. In the absence of a robust framework for evidence-based medicine, being vigilant and implementing a comprehensive, e.g. geriatric, model of care [30] are currently the most pragmatic ways of dealing with the uncertainties of managing multi-morbidity in ICU patients. The concept of comprehensive geriatric assessment can provide standardized screening and assessment tools for chronic conditions and disabilities [31]. Thereafter, a contribution by geriatricians to decisions about objectives and suitable levels of critical care can further support a holistic view and prevent inappropriate interventions. However, the specific approach to these challenges and, eventually, the outcome quality depend on the structure and workflows of the healthcare organisation [32].

The decision-making about tailoring critical care for multi-morbid individuals, especially in the very old, require consideration and weighting of patient-centred outcome measures such as quality of life vs burden of treatment [33]. Short- and long-term goals should be determined by the expectations of the individual patient, which may differ from recommendations for managing single conditions in younger people. However, a recent review was unable to identify methodologically robust studies about understanding personal preferences of multi-morbid patients presenting with acute diseases [34]. Thus, a precise adjustment of critical care to the personal needs of these individuals still remains elusive.

### Multi-morbidity and prognostication

Pre-admission characteristics, notably past trajectories of overall health, are known to be at least as important for predicting long-term outcome of critical care as the severity of the acute illness. Frailty and functional disabilities [35] are regarded as both long-term consequences of multi-morbidity [36, 37] and predictors for post-ICU outcome including functional status [16, 38]. In fact, frailty, as a measure of reduced resilience to physical stress, was discussed as the link between advanced multi-morbidity and increased mortality [39]. During the COVID-19 pandemic, multi-morbidity, frailty as well as the severity of the acute condition were all strong predictors of in-hospital death [40]. A recent study showed the additive role of functional disabilities for mortality in very old multi-morbid individuals [41]. Although multi-morbidity, frailty and functional disabilities overlap in many older patients [42, 43], there are individuals with multi-morbidity who cannot be classified as frail or disabled. This indicates the existence of distinct patterns of vulnerability among multi-morbid patients, which may benefit from new and different treatment approaches in critical care [8]. Importantly, multi-morbidity patterns and outcome are influenced by socioeconomic and ethnic factors which was highlighted by the COVID-19 pandemic [44].

## Conclusions

**Fig. 2:**
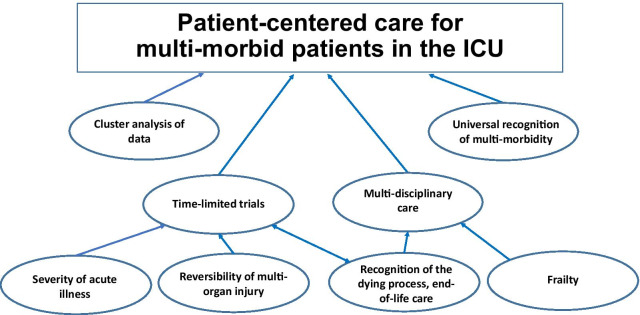
The elements of tailored and patient-centred critical care for multi-morbid patients.

What needs to be done to provide tailored and patient-centred critical care to multi-morbid patients? (Fig. 2). Firstly, multi-morbidity requires universal recognition in healthcare institutions historically structured to treat single conditions. Including multi-morbidity, frailty and functional status into the pre-operative assessment for post-operative prognostication and care planning was already suggested a decade ago [45]. Secondly, the (cluster) analysis of data obtained in realistic scenarios will help to develop quantitative, individually precise and, thus, clinically useful concepts of multi-morbidity. In combination with biomarkers for organ failure, that approach can give rise to composite risk prediction scores. The 'where' and 'when' of interventions should be defined by a more granular analysis of patients' trajectories in critical care. However, we have to pay attention to geographic and cultural characteristics of medical care [46]. Thirdly, we should deal with medical uncertainties with time-limited trials, where multi-morbid patients are admitted to ICU with patient-centred goals and clearly defined limitations concerning treatment escalation to enable the initiation of end of life care if necessary [47]. This framework also provides the opportunity to obtain longitudinal data, i.e. time series of observations, for a more precise predictive modelling [48]. Fourthly, long-term and patient-centred outcome crucially depends on post-ICU care, which should be planned and managed in a multi-disciplinary way, involving geriatricians as well as caregivers in the community [30]. Although much work still needs to be done, critical care can be better prepared for the coming wave of multi-morbid very old intensive care patients by tackling these issues [49].

## Data Availability

This is a viewpoint paper with no study data.
